# Association between new anthropometric indices and osteoporosis in Chinese postmenopausal women- retrospective study based on hospitalized patients in China

**DOI:** 10.3389/fendo.2025.1535540

**Published:** 2025-05-16

**Authors:** Xin Zhao, Jianbin Sun, Sixu Xin, Xiaomei Zhang

**Affiliations:** Department of Endocrinology, Peking University International Hospital, Beijing, China

**Keywords:** osteoporosis, lipid accumulation product, abdominal volume index, body roundness index, Chinese visceral adiposity index

## Abstract

**Objective:**

This study aimed to investigate the correlation between new anthropometric indicators and osteoporosis in postmenopausal women, evaluate whether these indicators can be used for the screening of osteoporosis in postmenopausal women, and provide evidence for the prevention of osteoporosis and fractures in these patients.

**Methods:**

This study retrospectively analyzed 470 females hospitalized in the Department of Endocrinology of Peking University International Hospital between January 2017 and August 2022. According to the bone mineral density (BMD) results, the subjects were divided into two groups: the normal group and the osteoporosis group.

**Results:**

(1) Compared with the normal group, the women in the OP group were older, the levels of BMD were lower, and the levels of 25(OH)D were lower, with all the differences being statistically significant (p<0.05). The body shape index (ABSI), Chinese visceral fat index (CVAI), abdominal volume index (AVI), and body roundness index (BRI) were significantly higher, and the body mass index (BMI) was significantly lower in the Osteoporosis (OP) group than in the normal group (all p<0.05). (2) CVAI was negatively correlated with hip BMD and lumbar spine BMD (r=-0.35, p<0.05; r=-0.20, p<0.05). BRI was negatively correlated with hip and lumbar spine BMD (r=-0.37, p<0.05; r=-0.20, p<0.05). (3) After adjusting age, blood pressure (BP), blood glucose, blood lipids, estimated Glomerular Filtration Rate (eGFR) and Ca levels, high levels of CVAI, AVI and BRI were independent risk factors for OP (OR=4.27, 95%CI 2.49, 7.33; OR=2.08, 95%CI 1.23, 3.51; OR=6.11, 95% CI 3.39, 11.01). (4) The model for predicting the risk of OP using anthropometric indicators showed that the AUCs ranked CVAI > BRI > ABSI > AVI = BMI > lipid accumulation index (LAP) > waist-to-hip ratio (WHR) > waist-to-height ratio (WHtR).

**Conclusion:**

This clinical study showed that new anthropometric indicators are associated with osteoporosis in postmenopausal women. It is necessary to pay attention to CVAI, BRI, AVI, and other anthropometric indicators in postmenopausal women, which are also of great significance for the prevention of osteoporosis.

## 1 Introduction

Osteoporosis is characterized by reduced bone mass and microstructural damage to the bone tissue, leading to increased bone fragility and susceptibility to fractures. Previous studies have confirmed that a low body mass index (BMI) is a risk factor for osteoporosis (1). However, in recent years, an increasing number of studies have demonstrated a protective effect of body mass on bone health is being questioned. Many studies have found that visceral fat is related to changes in bone microstructure, which has triggered a new direction for studying the effects of different types of obesity on bone health in different populations (2). According to the distribution site, it can be divided into visceral adipose tissue and subcutaneous adipose tissue, and excessive accumulation of visceral fat has the greatest impact on human health.

Studies have shown that an imbalance in adipokines secreted by visceral adipose tissue is involved in the occurrence of metabolic syndrome, diabetes, and osteoporosis (3). The influence of obesity on bone metabolism involves an increase in mechanical load, fat distribution, cytokine pathways, and bone marrow adipose tissue (4).

At present, the traditional body surface measurements commonly used to assess obesity include BMI, which reflects the degree of overall obesity, and waist circumference (WC), which assesses abdominal obesity. However, these traditional body surface measurements have limitations in the assessment of obesity. For example, BMI cannot be used to distinguish between fat and musculoskeletal disorders. They may contribute more to the body mass. Clinical and large-scale epidemiological studies mainly use anthropometric indicators such as WC and waist-to-hip ratio (WHR) to evaluate abdominal fat deposition; however, WC cannot distinguish differences caused by height differences, nor can it distinguish between visceral adipose tissue and subcutaneous adipose tissue (5). Therefore, bioelectrical impedance analysis, CT, MRI, and other techniques are used to evaluate the abdominal fat of patients, but these are expensive and not suitable for clinical screening evaluation. Therefore, different anthropometric indicators have been developed.

Therefore, scholars in China and abroad have proposed new body surface measurement indicators, such as the A body shape index (ABSI), lipid accumulation index (LAP), and visceral fat index (VAI). Krakauer et al. (6) developed an anthropometric index that combines WC, BMI, and height. Previous studies have confirmed that ABSI is correlated with abdominal adipose tissue and significantly correlated with mortality. The BRI is an anthropometric index proposed by Thomas et al. in 2013 (7), which can be calculated using waist circumference and height to evaluate body fat content, especially visceral fat. A higher BRI value indicated greater visceral fat deposition. The LAP (8) and VAI (9) can more accurately reflect the degree of body lipid accumulation and visceral fat content. Because the fat distribution characteristics of the Asian population are different from those of the European population, Xia et al. (10) further used a binary linear logistic regression model to construct the Chinese visceral fat index (CVAI) on the basis of VAI and the fat distribution characteristics of the Asian population.

The purpose of this study was to investigate the correlation between new anthropometric indicators and osteoporosis in postmenopausal women and to evaluate whether these indicators can be used for the screening of osteoporosis in postmenopausal women to provide new evidence-based medical evidence for the early detection and diagnosis of osteoporosis and prevention of the occurrence and development of osteoporosis and fractures in these patients.

## 2 Research subjects and methods

### 2.1 Ethics statement

The study was approved by the Ethics Committee of the Peking University International Hospital. The study was a retrospective analysis; therefore, the requirement for written informed consent was waived.

### 2.2 Research subjects

A total of 470 postmenopausal women with an average age of 64.23 ± 7.89 years old (50-80 years old) in the Department of Endocrinology between January 2017 and December 2022 were retrospectively analyzed. Exclusion criteria: (1) non-physiological postmenopausal women; (2) concomitant diseases that may cause secondary osteoporosis, such as endocrine (hyperparathyroidism syndrome, Cushing syndrome, hypogonadism, hyperthyroidism syndrome, prolactinoma, hyperprolactinemia, etc.), hematological diseases, connective tissue diseases (rheumatoid arthritis, lupus erythematosus, etc.), and chronic renal failure; (3) patients with a history of primary or secondary bone malignant tumors; and (4) patients who had used drugs for OP (estrogen bisphosphonates, active vitamin D, etc.).

### 2.3 Methods

#### 2.3.1 Clinical conditions

The following clinical data of each patient’s medical history were recorded: age, height, weight, blood pressure, systolic blood pressure (SBP), diastolic blood pressure (DBP), hip circumference (HC), and WC. BMI = Weight (kg)/Height (m^2^).

#### 2.3.2 Laboratory biochemical indices

Laboratory biochemical indices included fasting blood glucose (FBG), glycosylated hemoglobin (HbA1c), total cholesterol (TC), triglyceride (TG), low-density lipoprotein cholesterol (LDL-C), high-density lipoprotein cholesterol (HDL-C), serum creatinine (sCr), uric acid (UA), calcium (Ca), and parathyroid hormone (PTH) were collected from patients. Bone metabolism markers, including osteocalcin (OC), β-C-terminal cross-linked peptide (β-CTX), procollagen 1 N-terminal telopeptide (P1NP), and 25-hydroxyvitamin D (25 [OH] D), were also detected in the clinical laboratory.

#### 2.3.3 Bone mineral density measurement

A Discovery QDR SERIES dual-energy X-ray bone density instrument (Hologic, USA) was used to determine bilateral hip (including the femoral neck, trochanter, inside hip, Ward’s triangle, and total hip) and lumbar vertebral (L1-4) BMD. Quality control assessments were conducted daily using the instrument. The coefficient of variation is 1.0%. T scores were determined automatically by the instrument’s software using the Asian population as a reference (11). OP is defined by the WHO as a T score of ≤ –2.5 SD at any site, with osteopenia defined as –1.0 ≥ T score ≥ –2.5. The T-scores of the patients were used to establish three patient groups: normal, osteopenia, and OP.

#### 2.3.4 Anthropometric indicators

The formula for calculating the anthropometric indicators is as follows:

1. Body mass index (BMI)=weight(kg)/height²(m²)
2. Waist-to-height ratio (WHtR)=WC (cm)/height (cm)
3. Wasit-to-hip ratio (WHR) =WC (cm)/HC (cm)
4. Lipid accumulation product (LAP) (female) = [WC (cm) -58] ×TG
5. A body shape index (ABSI)= WC(m)/[BMI^2/3^×height(m)^1/2^]
6. CVAI (female)=-187.32 + 1.71×age+4.32×BMI+1.12×WC+39.76×log_10_TG-11.66×HDL-C
7. Abdominal volume index (AVI)= [WC^2^ (cm) +0.7× (WC-HC)^2^ (cm)]/1000
8. Body roundness index (BRI)=364.2-365.5× [1-π^-2^× WC^2^ (m) ×height^-2^ (m)]

### 2.4 Statistical analysis

The data were analyzed using the Kolmogorov-Smirnov test, and all variables had a normal distribution and were expressed as the mean ± standard deviation. Multi-group comparisons of the sample mean were compared with the one-way analysis of variance (ANOVA). Univariate and multivariate analyses of factors were performed using an unconditional logistic regression model, and the odds ratio (OR) and 95% confidence interval (CI) were calculated. The Receiver Operating Characteristic (ROC) curve was used to calculate the Area Under the ROC curve (AUC). All statistical tests were two-sided, with statistical significance set at p<0.05. Statistical analysis was conducted using SPSS version 22.0 software (IBM, Chicago, Illinois, USA).

## 3 Results

*1. ABSI, CVAI, AVI, and BRI were significantly higher and BMI was significantly lower in the OP group than in the normal group and osteopenia group*

Compared with the normal group, the women in the OP group and osteopenia were older, the BMD levels were lower, and the levels of 25(OH)D were lower, all of which were statistically significant (p<0.05). ABSI, CVAI, AVI, and BRI were significantly higher and BMI was significantly lower in the OP group than in the other two groups (all p<0.05). There were no significant differences in WHtR, LAP, and WHR among the three groups. There were no significant differences in blood pressure, blood lipid levels, eGFR, and bone metabolism markers among the three groups (p>0.05) (**Table 1**).

**Table 1:** Comparison of biochemical indice, BMD, bone metabolic markers, and anthropometric indexes among the three groups.

Index	Normal group (n=170)	Osteopenia (n=102)	Osteoporosis group (n=198)	F	p
Age (year)	62.89 ± 6.56	63.02 ± 6.59	64.72 ± 7.91	1.08	0.21
WC (cm)	89.70 ± 11.23	88.34 ± 9.02	91.05 ± 10.50	0.33	0.60
HC (cm)	96.83 ± 7.46	99.76 ± 5.98	99.02 ± 8.17	0.66	0.50
SBP (mmHg)	133.69 ± 14.66	137.47 ± 12.09	134.96 ± 17.41	0.11	0.89
DBP (mmHg)	77.67 ± 9.76	79.36 ± 8.87	77.05 ± 10.14	0.10	0.93
HbA1c (%)	8.53 ± 1.70	8.98 ± 1.76	8.53 ± 1.84	1.98	0.10
FBG (mmol/L)	8.70 ± 2.48	8.70 ± 3.47	8.69 ± 3.33	0.09	0.88
TC (mmol/L)	4.09 ± 1.09	4.34 ± 1.21	4.40 ± 1.11	0.55	0.55
TG (mmol/L)	1.84 ± 1.04	1.89 ± 0.99	1.84 ± 1.13	0.80	0.46
LDL-C (mmol/L)	2.62 ± 0.78	2.50 ± 0.80	2.56 ± 0.85	0.40	0.60
HDL-C (mmol/L)	1.01 ± 0.35	1.03 ± 0.24	1.04 ± 0.32	0.80	0.48
UA (mmol/L)	333.76 ± 79.34	340.34 ± 73.76	315.49 ± 88.21	1.24	0.39
eGFR (ml/min/1.73^2^)	89.53 ± 14.34	92.58 ± 13.47	90.99 ± 17.23	1.76	0.30
Ca(mmol/L)	2.29 ± 0.08	2.31 ± 0.09	2.30 ± 0.09	0.55	0.86
PTH	37.57 ± 14.79	37.09 ± 13.87	36.16 ± 14.55	0.89	0.56
Lumbar spine BMD(g/cm^2^)	0.99 ± 0.19	0.86 ± 0.14	0.76 ± 0.13	10.49	<0.05
Hip BMD(g/cm^2^)	0.89 ± 0.32	0.76 ± 0.20	0.56 ± 0.09	14.56	<0.05
OC (ng/ml)	14.68 ± 7.98	14.56 ± 4.90	14.22 ± 6.78	1.16	0.87
Beta CTX (ng/ml)	0.39 ± 0.20	0.44 ± 0.40	0.46 ± 0.49	1.66	0.32
P1NP (ng/ml)	45.80 ± 26.09	46.27 ± 23.54	44.17 ± 20.30	1.58	0.63
25(OH)D(ng/ml)	18.87 ± 6.90	15.87 ± 6.88	13.22 ± 6.08	2.87	<0.05
WHtR	0.57 ± 0.06	0.57 ± 0.08	0.57 ± 0.07	0.21	0.90
LAP	56.66 ± 30.94	54.09 ± 35.87	54.37 ± 36.05	0.69	0.42
ABSI	0.08 ± 0.01	0.08 ± 0.02	0.09 ± 0.02	5.70	<0.05
CVAI	4.32 ± 3.09	5.98 ± 4.09	7.24 ± 5.25	6.78	<0.05
AVI	8.48 ± 2.00	8.66 ± 2.09	8.94 ± 2.05	2.12	<0.05
BRI	4.08 ± 1.45	4.98 ± 1.09	5.84 ± 1.98	5.00	<0.05
WHR	0.94 ± 0.07	0.93 ± 0.07	0.93 ± 0.07	1.08	0.29
BMI	25.66 ± 3.52	25.33 ± 3.28	25.03 ± 3.37	1.91	<0.05

WC, waist circumference; HC, hip circumference; SBP, systolic blood pressure; DBP, diastolic blood pressure; BMI, body mass index; FBG, fasting blood glucose; HbA1c, glycosylated hemoglobin; UA, uric acid; Ca, calcium; TC, total cholesterol; TG, triglyceride; LDL-C, low-density lipoprotein cholesterol; HDL-C, high-density lipoprotein cholesterol; PTH, parathyroid hormone; OC, osteocalcin; β-CTX, beta C-terminal telopeptide; P1NP, procollagen 1 intact N-terminal; 25(OH)D, 25-hydroxyvitamin D; eGFR, glomerular filtration rate; BMD, bone mineral density; BMI, body mass index; WHtR, waist-to-height ratio; CVAI, Chinese visceral adipose index; BRI, body roundness index; AVI, abdominal volume index; ABSI, body shape index; LAP, lipid accumulation product; WHR, waist-to-hip ratio.

*2. CVAI was negatively correlated with hip and lumbar spine BMD*

The results of the correlation analysis showed that CVAI was negatively correlated with hip and lumbar spine BMD (r=-0.35, r=-0.20, p<0.05). BRI was negatively correlated with hip BMD and lumbar spine BMD (r=-0.37, r=-0.20, p<0.05) (**Table 2**).

**Table 2:** Correlation analysis between BMD and anthropometric indicators.

Index	Hip BMD		Lumbar Spine BMD	
	r	p	r	p
WHtR	0.04	0.38	0.14	<0.05
LAP	-0.01	0.93	0.10	<0.05
ABSI	-0.20	<0.05	-0.07	0.10
CVAI	-0.35	<0.05	-0.20	<0.05
AVI	-0.14	<0.05	-0.04	0.35
BRI	-0.37	<0.05	-0.20	<0.05
WHR	0.01	0.94	0.04	0.40
BMI	0.09	0.06	0.07	0.11

BMI, body mass index; WHtR, waist-to-height ratio; CVAI, Chinese visceral adipose index; BRI, body roundness index; AVI, abdominal volume index; ABSI, body shape index; LAP, lipid accumulation product; WHR, waist-to-hip ratio.

*3. High levels of CVAI, AVI and BRI were independent risk factors for OP*

Logistic regression analysis was performed with OP as the dependent variable and anthropometric indicators as independent variables. The results showed that, after adjusting age, blood pressure, blood glucose, blood lipids, eGFR and Ca, high levels of CVAI, AVI and BRI were independent risk factors for OP (OR=4.27, 95% CI 2.49, 7.33; OR=2.08, 95%CI 1.23, 3.51; OR=6.11, 95%CI 3.39, 11.01) (**Table 3**).

**Table 3:** Logistic regression analysis of anthropometric indicators and OP.

Index	Crude OR	95%CI	P	Adjust OR	95%CI	p
WHtR						
Low	1			1		
Medium	0.92	0.59, 1.43	0.70	1.16	0.69, 1.93	0.58
High	10.92	0.59, 1.43	0.70	0.85	0.50, 1.44	0.55
LAP						
Low	1			1		
Medium	1.27	0.82, 1.98	0.29	1.12	0.68, 1.85	0.66
High	0.85	0.55, 1.34	0.49	0.73	0.43, 1.23	0.24
ABSI						
Low	1			1		
Medium	1.19	0.75, 1.86	0.46	1.16	0.68, 1.97	0.58
High	1.71	1.10, 2.68	<0.05	1.56	0.93, 2.62	0.09
CVAI						
Low	1			1		
Medium	1.28	0.79, 2.06	0.31	1.25	0.72, 2.71	0.42
High	4.23	2.64, 6.75	<0.05	4.27	2.49, 7.33	<0.05
AVI						
Low	1			1		
Medium	1.10	0.70, 1.74	0.67	1.17	0.70, 1.98	0.55
High	1.78	1.14, 2.79	<0.05	2.08	1.23, 3.51	<0.05
BRI						
Low	1			1		
Medium	1.07	0.66, 1.72	0.79	1.66	0.95, 2.91	0.08
High	3.48	2.20, 5.51	<0.05	6.11	3.39, 11.01	<0.05
WHR						
Low	1			1		
Medium	0.81	0.47, 1.37	0.42	0.79	0.43, 1.43	0.44
High	0.66	0.37, 1.16	0.15	0.73	0.38, 1.37	0.32
BMI						
Low	1			1		
Medium	0.71	0.45, 1.13	0.15	0.71	0.42, 1.21	0.20
High	0.58	0.37, 0.92	<0.05	0.56	0.33, 0.96	<0.05

BMI, body mass index; WHtR, waist-to-height ratio; CVAI, Chinese visceral adipose index; BRI, body roundness index; AVI, abdominal volume index; ABSI, body shape index; LAP, lipid accumulation product; WHR, waist-to-hip ratio.

*4. The corresponding cut-off point of CVAI for predicting OP was 5.89*

**Figure 1:**
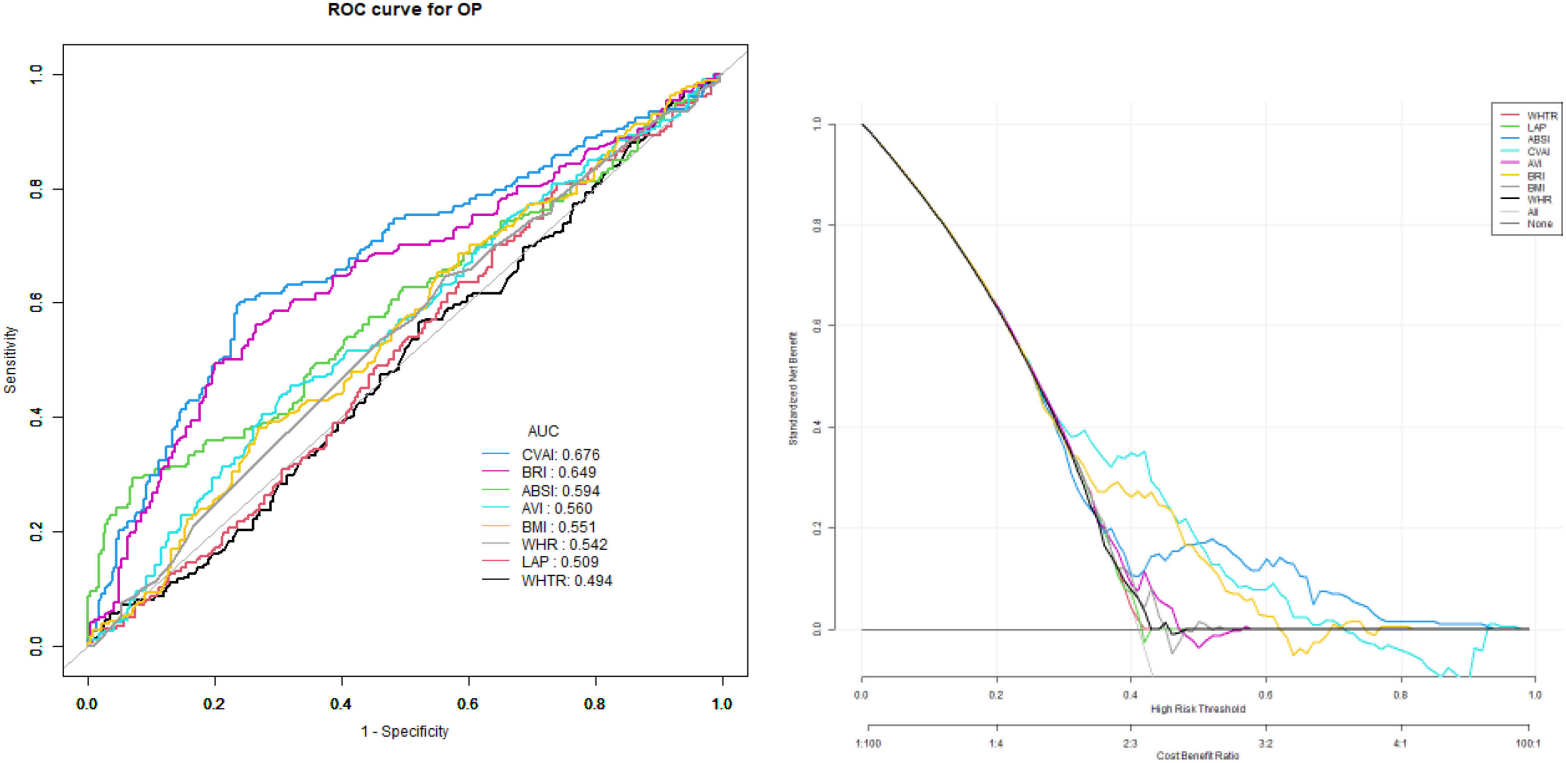
The models used to predict the risk of OP using anthropometric indicators showed that the AUC of the models ranked CVAI>BRI>ABSI> AVI= BMI>LAP>WHR>WHtR.

The model used to predict the risk of OP using anthropometric indicators showed that the AUC of the models ranked CVAI>BRI>ABSI> AVI= BMI>LAP>WHR>WHtR. The corresponding cutoff point of CVAI for predicting OP was 5.89 (**Table 4**, **Figure 1**).

**Table 4:** Univariate predictive models of OP with anthropometric indicators.

Index	AUC	95%CI	Cut-off point	Specificity	Sensitivity
WHtR	0.50	0.44, 0.55	0.56	0.48	0.57
LAP	0.51	0.46, 0.56	76.84	0.26	0.81
ABSI	0.61	0.56, 0.66	0.10	0.93	0.30
CVAI	0.66	0.61, 0.71	5.89	0.76	0.60
AVI	0.55	0.50, 0.60	9.19	0.68	0.45
BRI	0.64	0.59, 0.69	5.60	0.74	0.56
BMI	0.55	0.50, 0.60	23.62	0.73	0.38
WHR	0.52	0.47, 0.59	0.94	0.44	0.65

BMI is for body mass index, WHtR, waist-to-height ratio; CVAI, Chinese visceral adipose index; BRI, body roundness index; AVI, abdominal volume index; ABSI, a body shape index; LAP, is for lipid accumulation product; WHR, waist-to-hip ratio.

## 4 Discussion

Previous studies have confirmed that body mass index (BMI) is a protective factor against osteoporosis. Increases in body weight and BMI can reduce the risk of OP, and the mechanical load of body weight can stimulate bone growth. However, compared to BMI, recent studies have focused on the relationship between visceral fat and bone health. Some studies have proposed that visceral fat is a risk factor for osteoporosis (12), and a higher level of visceral adipose tissue is harmful to bone health. BMI cannot assess whole-body fat content; therefore, it has certain clinical limitations to rely on BMI alone to assess the risk of OP. Therefore, it is necessary to explore more indicators of visceral fat accumulation and anthropometric indicators to assess the risk of OP in postmenopausal women. The results of this study showed that CVAI was negatively correlated with hip and lumbar spine BMD, and BRI was negatively correlated with hip and lumbar spine BMD.

This study further established a logistic regression analysis with OP as the dependent variable and anthropometric indicators as independent variables, and the results showed that, after adjusting age, blood pressure, blood glucose, blood lipids, eGFR and Ca levels, high levels of CVAI, AVI and BRI were independent risk factors for OP (OR=4.27, 95% CI 2.49, 7.33; OR=2.08, 95%CI 1.23, 3.51; OR=6.11, 95%CI 3.39, 11.01).

The mechanism of the correlation between obesity and osteoporosis and fracture risk mainly includes the following aspects: (1) mechanical load: weight gain increases the mechanical load of bones, which can activate osteocytes and dendritic cells and promote the expression of insulin-like growth factor-1 and osteocalcin (13). (2) adipocytokines: adipocytokines secreted by adipocytes include leptin, adiponectin, and resistin. Studies have found that leptin receptors are present on the surfaces of osteoblasts and chondrocytes (14). Leptin can inhibit osteoclast differentiation, promote osteoblast proliferation and bone mineralization, and promote collagen synthesis (15). Serum leptin levels were positively correlated with BMI and the total amount of adipose tissue. Compared to normal-weight individuals, the serum leptin level in obese individuals is significantly higher (16), and the BMD of the lumbar spine is positively correlated with leptin levels (17). Adiponectin stimulates the proliferation and differentiation of osteoblasts and inhibits the activation and differentiation of osteoclasts by increasing alkaline phosphatase, osteocalcin, and type I collagen levels, which are conducive to bone formation (18). However, other studies have found that adiponectin inhibits the expression of osteoprotegins by activating the receptor activator for nuclear factor-κ B ligand (RANKL), which has adverse effects on bone health. Studies have shown that adiponectin is associated with lower BMD (19). At present, results of studies on the effect of adiponectin on OP are inconsistent, and further studies are needed to explore the effect of adiponectin on bone health. (3) Inflammatory factors, including many inflammatory factors and adipokines, are also involved in the development of osteoporosis. Individuals with obesity exhibit a low-grade inflammatory phenotype. Inflammatory factors released during inflammation increase bone resorption and inhibit bone formation by activating osteoclast activity. Several studies have shown that the pro-inflammatory factors interleukin-6 (IL-6) and tumor necrosis factor α (TNF-α) promote osteoclast maturation and activate osteoclast activity (20), and the inflammatory factors released by inflammation also affect bone microstructure. The risk of hip fracture in women is related to the soluble receptors IL-1 and TNF-α (21). TNF-α promotes osteoclast formation by activating RANKL. IL-6 mRNA is expressed in osteoclast precursor cells and osteoclasts and can promote the formation of osteoclasts and bone resorption. (4) Influence of key signaling pathways: Lipid metabolism disorders can also affect osteoblast differentiation by interfering with key signaling pathways (22). Adipocytes secrete cytokines that regulate the state of the bone. Studies have shown that the higher the proportion of fat in the bone marrow, the lower the density of bone trabeculae. Aromatase produced by adipocytes is one of the main sources of aromatase in the human body, which can convert androstenedione produced by the adrenal gland into estrone and increase estrogen levels. Estrogen is a steroid hormone that prevents OP by reducing bone resorption and stimulating bone formation.The VAI is a sex-specific index of visceral adiposity based on BMI, WC, TG, and HDL-C levels. Studies have found that VAI can reflect the endocrine function and low-grade inflammatory state of adipose tissue to a certain extent and is a simple tool to assess the risk of cardiovascular disease in people without obvious metabolic syndrome (23). Presently, the results of domestic and foreign studies on the correlation between VAI and OP are inconsistent. Tian et al. (24) showed that, consistent with BMI and WC, the risk of osteoporosis decreased with the increase of VAI. A recent study based on the NHANES indicated that an increased VAI is independently linked to a higher prevalence of osteoporosis among older adults in the US. Further analysis revealed that once VAI reaches a certain threshold, femur BMD no longer increases and may even decrease (25). Another study also showed that in postmenopausal women, the relationship between VAI and BMD is nonlinear (U-shaped) (26). In our study, the results showed that CVAI was significantly negatively correlated with BMD (P <0.05).

The results of this study showed that CVAI was negatively correlated with BMD and, after adjusting age, blood pressure, blood glucose, blood lipids, eGFR and Ca levels, high level of CVAI was an independent risk factor for OP (OR=4.27, 95%CI 2.49, 7.33). Previous studies were based on European and American populations, but there is a lack of studies on Asian populations, and the fat distribution of Asian populations is different from that of European and American populations. Therefore, we chose the CVAI, an indicator that is more suitable for the Chinese population, which may be the main reason for the inconsistent results of various studies. Our results showed that the risk of OP was significantly increased only at a high level of CVAI and that CVAI was not a risk factor for OP at a moderately elevated level. However, this result needs to be verified in a larger population in future studies. In addition, this study further analyzed the cutoff point of OP, and the CVAI for predicting OP was 5.89. In addition, VAI was found to have the strongest predictive power for OP among all the anthropometric indices.The AVI is an anthropometric index calculated based on the HC and WC proposed by Fernado (27), and is used to estimate the total abdominal cavity volume, theoretically including the volume of intra-abdominal adipose tissue. A cross-sectional study showed that AVI is a reliable anthropometric tool and that the estimation of visceral fat by abdominal cavity volume is closely related to IGT and T2DM. At present, there are few studies on the correlation between the AVI and OP in China and abroad. A recent study showed that in postmenopausal women, the relationship between VAI and BMD is nonlinear (U-shaped) (26). The results of this study showed that AVI was negatively correlated with hip BMD, and a high level of AVI was an independent risk factor for OP (OR=2.08, 95%CI 1.23, 3.51), but the predictive value of AVI for OP was lower than that of CVAI.The BRI is an anthropometric index proposed by Thomas et al. in 2013 (7), with the aim of more accurately measuring and evaluating the distribution of body fat and related health risks. BRI calculates an elliptical model based on body shape using eccentricities that estimate visceral fat and percentage of total body fat. Unlike traditional BMI, BRI considers weight as well as height, as well as important parameters such as WC. This allows the BRI to capture the distribution of visceral fat more fully than just total body fat. BRI has a wide range of clinical applications. Studies have shown that BRI is superior to other traditional anthropometric measures for assessing the risk of various clinical endpoints such as renal disease (28) and cardiometabolic disease (29). This means that the BRI can provide a more accurate assessment of fat distribution and more effective prediction and prevention of various serious obesity-related health problems.

The results of this study have showed that, BRI was negatively correlated with hip BMD and lumbar spine BMD, and after adjusting age, blood pressure, blood glucose, blood lipids, eGFR and Ca levels, high level of BRI was an independent risk factor for OP (OR=6.11, 95%CI 3.39, 11.01), and BRI had a certain predictive value for OP. This is consistent with a recent NHANES-based study that demonstrated a notable inverse relationship between BRI and total BMD, indicating that a higher BRI could be associated with a lower BMD and a potentially greater risk of developing OP (30). Although the populations in the two studies were ethnically different, the results were similar, further indicating that the relationship between BRI and BMD is inversely related.In 2012, Krakauer et al. (6) proposed ABSI, which uses BMI and height to correct WC. The higher the ABSI value, the larger the WC under certain weight and height conditions, which is more consistent with visceral obesity. Some studies have found that ABSI may be a visceral abdominal marker related to adverse metabolic changes, which can be used for the risk assessment of atherosclerotic disease in postmenopausal women. At present, the association between ABSI and osteoporosis reported in China and abroad has not reached a unified conclusion. The results of this study showed that the level of ABSI was higher in the OP group and that ABSI was negatively correlated with hip BMD. However, ABSI was not an independent risk factor for OP after adjusting for age, blood lipids, blood glucose, blood pressure, and other factors. Similar to the results of this study, Murat et al. (31) used dual-energy X-ray absorptiometry to obtain bone mineral density of the lumbar spine and femoral neck, and the analysis showed that ABSI was not correlated with bone mineral density. However, another recent study (32) showed that a higher ABSI increases the risk of osteoporosis independently and synergistically with low eGFR in elderly Chinese adults. Another study based on the NHANES showed (33) a significant negative correlation between ABSI and BMD at the four detection sites of the femur, and this correlation may vary slightly due to age, race, family income, and different detection sites. These results indicate that, compared to overall body weight, fat distribution and content may be more closely related to bone metabolism. Due to data limitations, this study did not further evaluate the correlation between ABSI and OP. Therefore, ABSI is a risk factor for bone mineral density loss, but further research is needed to determine whether ABSI is an independent risk factor for OP. Given that there are different formulas for ABSI calculation in different ethnic groups, the key to its application in research is to choose the appropriate formula for scientific research.

This study had a few limitations. First, the participants in this study were postmenopausal women and could not represent the results of all populations. We need to expand the sample size and include male populations to further reveal the correlation between CVAI and OP. Second, as this was a retrospective study, prospective research is needed to explore the potential mechanisms and pathways underlying the relationship between CVAI and OP. Finally, we need to further validate our results using animal experiments and explore the mechanisms underlying the correlation between CVAI and OP. Currently, research on the relationship between human measurement indicators and OP is limited, and our findings are only preliminary. In future studies, we will expand the sample size and conduct multicenter studies to improve the results of this study.

## 5 Conclusions

This clinical study showed that new anthropometric indicators are associated with osteoporosis in postmenopausal women. Although BMI is a protective factor against osteoporosis, attention should be paid to osteoporosis screening in postmenopausal women with excessive body weight and visceral fat accumulation. In clinical practice, CVAI, BRI and AVI should be paid attention to in postmenopausal women, which is of great significance for the prevention of osteoporosis.

## Data Availability

The raw data supporting the conclusions of this article will be made available by the authors, without undue reservation.
